# Case Report: Primary urethral repair in a cat secondary to urethral tear sustained during the catheterization procedure

**DOI:** 10.3389/fvets.2025.1481879

**Published:** 2025-03-21

**Authors:** Kimery L. Hankins, Laurie A. Zacher

**Affiliations:** ^1^Department of Small Animal Surgery, Central Texas Veterinary Specialty & Emergency Hospital, Round Rock, TX, United States; ^2^University of Missouri College of Veterinary Medicine and Surgery, Columbia, MO, United States

**Keywords:** cat, perineal urethrostomy (PU), urinary catheter, cystourethrogram, primary urethral repair

## Abstract

This report describes a case of primary urethral repair secondary to a urethral tear in a 4-year-old, male castrated, domestic shorthair cat. The cat was initially presented on an emergency basis for complete urethral obstruction with radiographic evidence of cystolithiasis. A perineal urethrostomy and concurrent cystotomy were performed to relieve the urethral obstruction and retrieve the uroliths. One day postoperatively, a uroabdomen secondary to a urethral tear was diagnosed. An indwelling Foley catheter was placed. A contrast cystourethrogram performed 4 days later, however, revealed a persistent urethral tear, so a Foley catheter was replaced. Seven days after replacement, another contrast cystourethrogram was performed revealing a persistent urethral tear. Due to the anatomic location of the tear identified on radiographs after the contrast study, primary closure of the urethral defect with the placement of a Foley urinary catheter was completed. Another contrast urethrogram 7 days after repair revealed a resolved defect with no leakage appreciated once the urinary catheter was removed. This case highlights the management, surgical techniques, and success of a primary urethral repair in a cat. It demonstrates successful outcomes and follow-up for an uncommon procedure and illustrates the importance of initial catheterization for blocked felines.

## 1 Introduction

Urethral obstruction is a commonly reported condition in veterinary medicine primarily in male cats in which the narrow distal urethra occludes due to a variety of disease processes (1). Feline idiopathic cystitis (previously known as feline lower urinary tract disease), ureterolithiasis, trauma, or stricture to the distal urethra, neoplasia, and idiopathic obstructions are a few conditions that can cause impediment (2–5). It is a condition that is typically emergent for the animal but easy to diagnose given history, signalment, physical examination, and often specific clinical signs associated with presentation (6). However, intervention involving the use of catheterization of the penile urethra can cause unforeseen trauma that can sometimes result in rupture or tear of the urethral mucosa (6, 7). In instances where the urethra is damaged beyond second intention healing using indwelling Foley catheters, surgical procedures such as a perineal urethrostomy, prepubic urethrostomy, transpelvic urethrostomy, or urethral anastomosis can occasionally be executed (1, 8, 9). However, as the risk of stoma stricture is higher after a perineal urethrostomy has been peformed (10), revision of the original perineal urethrostomy stoma or salvage procedures such as a prepubic or transpelvic urethrostomy may be required and not always feasible depending on the location of the defect (1, 5, 8). Complication rates following salvage procedures such as prepubic or transpelvic urethrostomy are also higher in comparison with perineal urethrostomy and associated with a perceived decrease in quality of life for the pet (8, 10). Therefore, urethral resection and anastomosis can be used, although prognosis is guarded due to the expected severe stenosis of the urethral lumen (1). To the author’s knowledge, no report has described the primary closure of a urethral defect cranial to the pelvis in a cat without attempted revision first.

This case report describes the clinical presentation, treatment, and successful outcome of primary urethral repair secondary to catheterization trauma in a cat. Long-term follow-up 8 weeks postoperatively is also included.

## 2 Case description

### 2.1 Clinical history

A 4-year-old, male castrated, 7.2 kg domestic shorthair cat was presented with a 1-day history of stranguria, vocalization, overgrooming, and emesis with no observed urination. The cat had no previous medical concerns, and the only surgical history was a routine castration performed by the primary care veterinarian several years prior.

The cat was initially brought to his primary care veterinarian, where he received supportive treatment including anti-inflammatory, analgesic, and IV fluids. A cystocentesis was performed to withdraw blood-tinged urine from which a sample was collected. Urinalysis revealed hematuria, crystalluria, and bacteriuria. Blood was submitted for a complete blood count and serum biochemical profile. The cat was non-azotemic and was noted to be mildly neutrophilic (10.88 × 10^3/uL; range, 2.3–10.29 × 10^3/uL) suspected to be an inflammatory response with no other changes noted clinicopathologically. The cat was sedated using buprenorphine (0.02 mg/kg) administered intramuscularly and masked isoflurane and a urinary catheter was attempted to be passed numerous times unsuccessfully to relieve the urethral obstruction. He was then transferred to a referral hospital for emergency care.

At the emergency service, the cat was sedated for urethral catheterization, indwelling urinary catheter placement, and abdominal radiographs. The cat was then hospitalized overnight for continued monitoring and treatment and then referred to the surgery service the following morning.

### 2.2 Physical examination

Upon presentation to Central Texas Veterinary Specialty & Emergency Hospital, the cat was quiet, alert, and responsive with normal vital parameters. He was assessed as 5% dehydrated and had a moderately sized, firm urinary bladder that was mildly painful on palpation. The remainder of the physical examination was within normal limits. A triage abdominal ultrasound was performed, which showed no free peritoneal fluid.

### 2.3 Diagnostic findings

**Figure 1:**
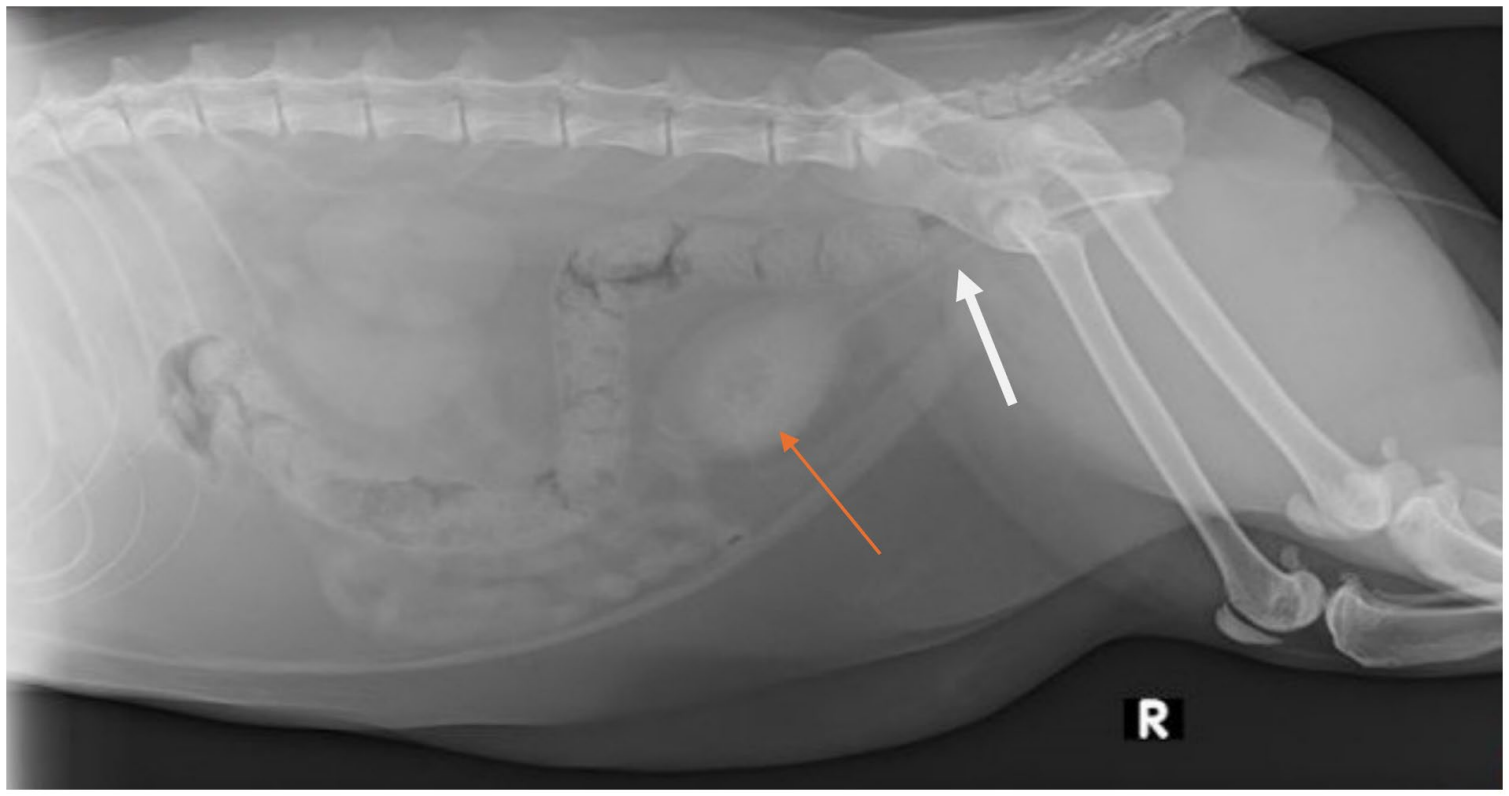
Right lateral abdominal radiograph obtained post-placement of an indwelling urinary catheter (white arrow) in a 4-year-old, castrated male, domestic shorthair cat that was evaluated for a 1-day history of lower urinary signs which progressed to complete urethral obstruction. There are numerous, small, mineral opacities that fill the lumen of the urinary bladder as indicated by the orange arrow.

Abdominal radiographs performed revealed numerous radiopaque urocystoliths (Figure 1).

Based on the diagnostic results and consistent clinical history, the cat was diagnosed with lower urinary tract obstruction and urinary tract infection secondary to urocystolithiasis, and surgical removal of the cystoliths along with a perineal urethrostomy (PU) was recommended.

### 2.4 Treatment and outcomes

The cat was premedicated and anesthesia induced to obtain a surgical plane of anesthesia. The cat was then positioned in dorsal recumbency and prepared for aseptic surgery. A routine perineal urethrostomy (PU) was performed following a modified Wilson–Harrison technique. Once completed, an approximately 5 cm caudoventral midline incision was made in the caudal abdomen to facilitate a routine cystotomy. Upon opening the abdomen, the urinary bladder was identified, and too numerous to count small cystoliths and debris were evacuated and flushed easily from the urinary bladder through the PU stoma normograde and retrograde using an 8Fr red rubber catheter. A culture of the urinary bladder mucosa and cystoliths retrieved was submitted for analysis. The bladder and abdomen were closed as per routine procedure, and liposomal bupivacaine (5.3 mg/kg) was infused into the tissues peri-abdominal incision. Postoperative radiographs were performed before recovery to confirm that no residual stones were present. The cat recovered from surgery without complications.

The following day, no urination had been noted since pulling the previously placed urinary catheter prior to surgery. Abdominal palpation revealed a small, soft urinary bladder, and point-of-care abdominal ultrasound demonstrated a large volume of free peritoneal fluid that was sampled and consistent with a uroabdomen (BUN >130 mg/dL ref. 16–36 mg/dL, creatinine too high to read ref. 0.8–2.4 mg/dL). A comparative, abbreviated panel of bloodwork was performed, which revealed mild azotemia (BUN 56 mg/dL ref. 16–36 mg/dL, creatinine 4.9 mg/dL ref. 0.8–2.4 mg/dL). Mild sedation was administered using ketamine (0.2 mg/kg IV) and midazolam (0.2 mg/kg IV), and a 5Fr indwelling Foley catheter was placed and maintained for 4 days from diagnosis of uroabdomen (5 days postoperatively).

**Figure 2:**
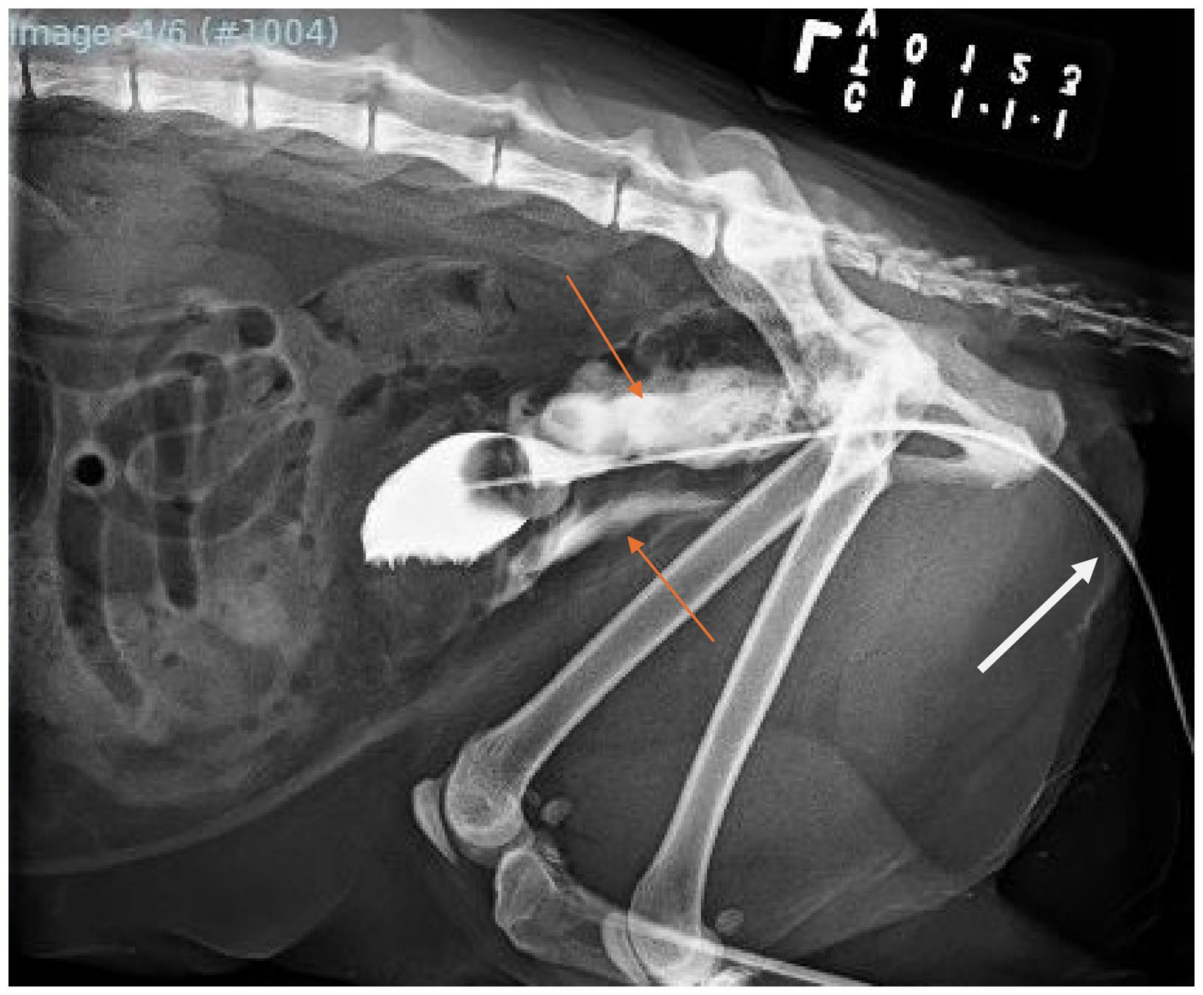
Left lateral caudal abdominal radiograph with retrograde positive-contrast cystourethrogram via an indwelling urinary catheter (white arrow) obtained 4 days post-conservative management of the diagnosed urethral tear. Note the severe extravasation of radiopaque contrast into tissues surrounding the urethra cranial to the pelvis (orange arrows).

**Figure 3:**
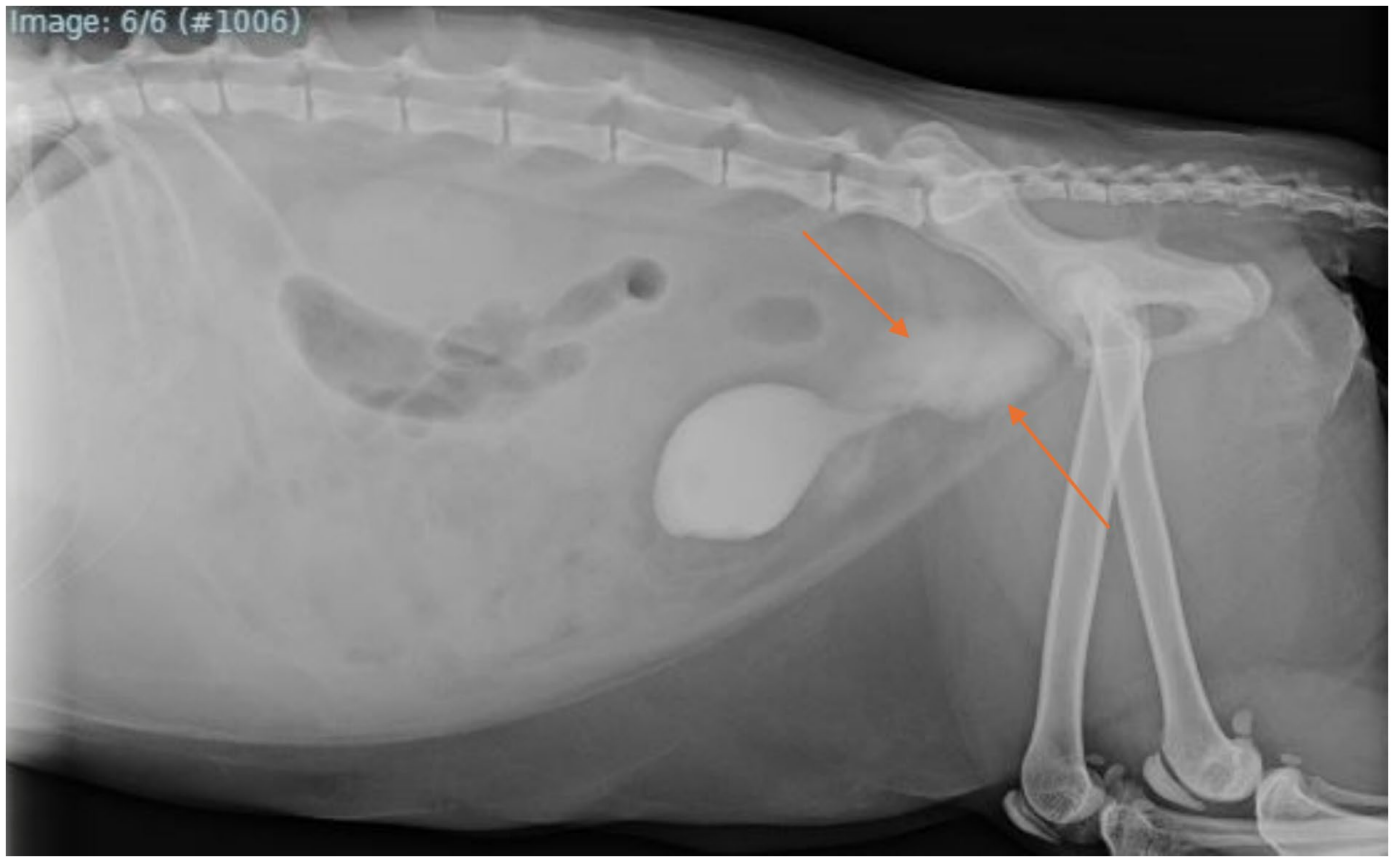
Right lateral abdominal radiograph with retrograde positive-contrast cystourethrogram obtained following an additional 8 days of conservative management for the persistent urethral tear. Note the persistent appearance of a severe amount of radiopaque contrast surrounding the urethra cranial to the pelvis (orange arrows).

On day 5 postoperatively, the urinary catheter was removed and a contrast cystourethrogram was performed, which revealed a urethral tear cranial to the pelvis (Figure 2). An 8Fr indwelling Foley catheter was replaced and maintained for an additional 7 days. During this time, the cat began having diarrhea, so he was started on metronidazole (10 mg/kg orally q12h) for a 4-day course. He additionally became hyperthermic suspect due to long-term catheterization and persistent bacterial infection, so meropenem (11 mg/kg SC q12h) was started and administered for a total of 13 days. Twelve days postoperatively, a repeat cystourethrogram was performed, which revealed a persistent urethral tear (Figure 3). Another 8Fr indwelling Foley catheter was placed and maintained for 3 days until a primary urethral repair could be performed.

On the day of primary urethral repair, the cat was again premedicated and anesthesia induced. The cat was positioned in dorsal recumbency and prepared for aseptic surgery. A caudoventral midline incision approximately 10 cm in length was made over the previous incision line. Upon entering the abdomen, multiple adhesions were observed and carefully broken down with monopolar electrocautery. The urinary bladder was isolated, and a stay suture of 4–0 poliglecaprone was placed at the apex. The urethra was identified and dissected until a 7 mm rent just cranial to the pubic bone was identified along the right dorsolateral surface. Primary closure using 6–0 glycomer 631 in a simple interrupted fashion was achieved and leak tested utilizing an 8Fr red rubber catheter, which revealed no additional urethral defects. An indwelling 8Fr Foley catheter was placed and maintained for 7 days following primary urethral repair.

**Figure 4:**
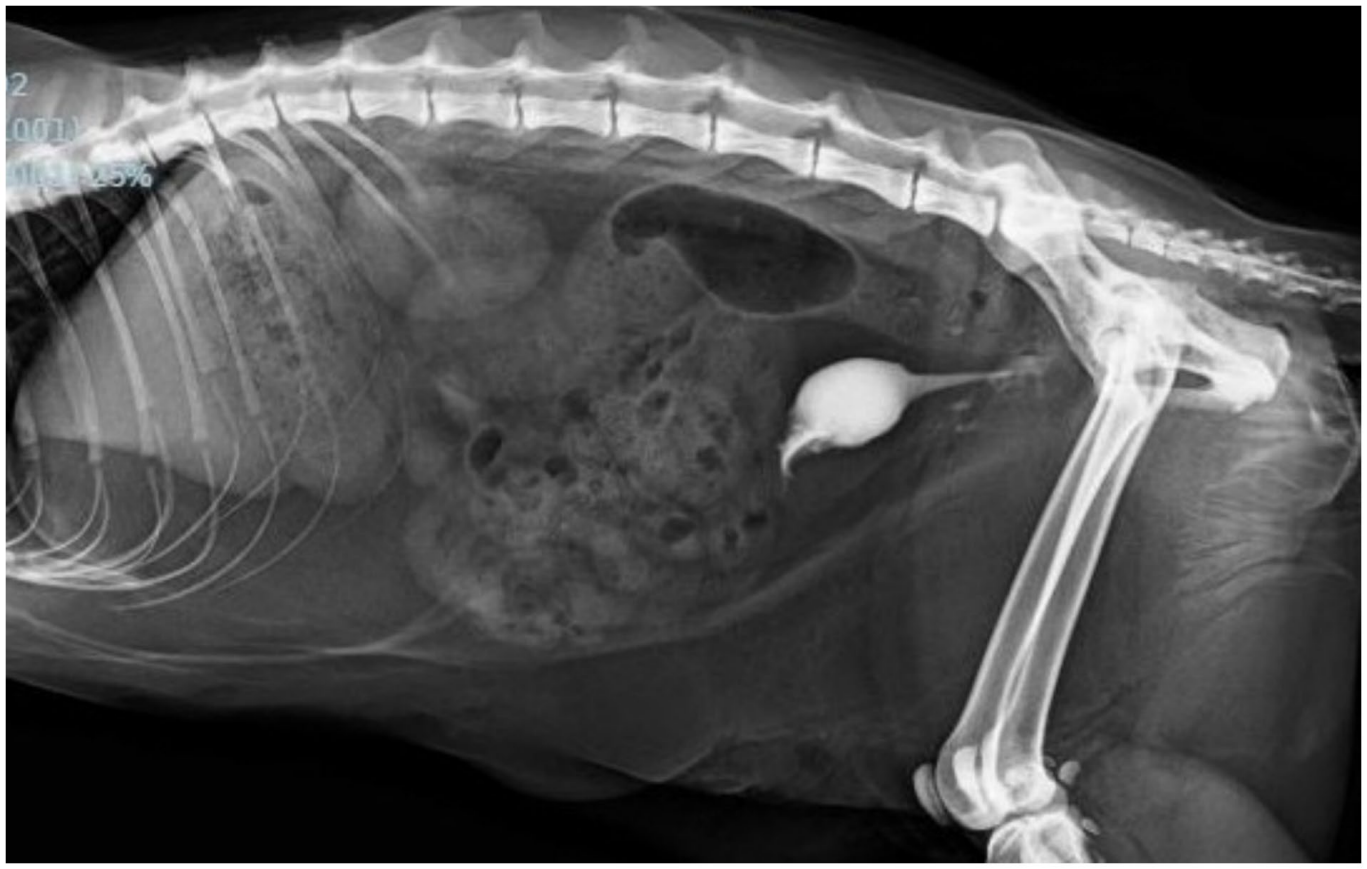
Left lateral abdominal radiograph with retrograde positive-contrast cystourethrogram obtained 7 days post-primary urethral tear repair. Note the continuous flow of radiopaque contrast between urethra into the urinary bladder with no extravasation appreciated surrounding the urethra as previously seen.

One week following primary repair, another cystourethrogram was performed, which demonstrated a resolved urethral tear (Figure 4), so the urinary catheter was removed and appropriate urination by the cat was observed. He was subsequently discharged later that afternoon.

At 2 weeks postoperatively following the primary urethral defect repair, the cat was noted to be doing well at home, urinating appropriately, and fully healed incisions assessed virtually.

Eight weeks postoperatively, the cat was assessed via email communication with the owner as still doing well but with sporadic demonstrations of lower urinary tract signs such as frequent urination and increased grooming of his perineal region. He was subsequently diagnosed with a urinary tract infection by the primary care veterinarian and treated accordingly.

## 3 Discussion

Primary urethral repair has been documented infrequently in veterinary medicine, with anastomosis of the defect the most commonly reported (11). In one study, graft material harvested from the urinary bladder was utilized to repair the urethral defect (12). Conservative management utilizing an indwelling urinary catheter to oppose urethral ends and allow for second intention healing has also been described often for cats with urethral tears or rupture (13). However, this is the first case report documenting the primary closure of a urethral tear cranial to the pelvis in a cat after a perineal urethrostomy had already been performed without further diversion of the remaining urethra. This is one of a multitude of cases reporting urethral trauma in cats following catheterization due to obstruction (7, 14) but the only describing the surgical procedure and successful outcome. Before this report, surgical revision typically involved a urethral anastomosis as reported in dogs (9, 15) or permanent urinary diversion in the form of perineal, prepubic, or transpelvic urethrostomy (8).

Primary urethral repair in cats specifically can be difficult, likely due to the small diameter of the feline urethra and complications resulting in stricture or dehiscence (16). In one article reviewing the outcomes of 63 cats following urethral rupture, 11% of cats underwent urethral anastomosis, with only about half surviving to discharge (17). However, this study did not discuss the anastomotic technique used nor the subsequent management of these cases to demonstrate the effectiveness of this type of repair. With regard to the present case, suturing over an indwelling urinary catheter to aid in the visualization of the urethral defect and leaving the catheter for an additional 7 days following repair likely contributed to the successful long-term outcome. Retention of the indwelling urinary catheter postoperatively not only prevented urinary extravasation which would delay wound healing but also hindered immediate urethral stricture at the repaired site.

Diagnosis of a urethral tear and subsequent uroabdomen, which can be categorized under primary abdominal compartment syndrome (18), is often achieved based on the entire clinical picture along with the use of various imaging modalities (19). Clinical signs such as anuria, lack of a palpable urinary bladder, and pain on abdominal palpation along with a history of attempted catheterization to alleviate urethral obstruction are a few elements that may indicate an issue (1, 19, 20). Diagnostic modalities such as initial ultrasound assessment of the abdomen, i.e. point of care ultrasound, or abdominal radiography can then be utilized to reveal free peritoneal fluid (19). Imaging findings can occasionally be subtle, however, especially if no free abdominal fluid is available to sample and corroborate as urine via biomechanical testing and cytology (1). Thus, one of the most common diagnostic tests performed, retrograde positive-contrast cystourethrography, can be used to highlight the lower urinary tract and identify defects as evidence of urethral tearing if the contrast material extravasates into surrounding tissues (20). Advanced imaging such as fluoroscopy or computed tomography (CT) are also options that can identify urethral defects (1, 19). In this particular case, positive-contrast cystourethrograms indicated persistent urethral tearing following conservative management and allowed for appropriate planning and management of primary urethral repair based on the location of extravasated contrast material cranial to the pelvis. Understanding the mechanism and pathophysiology of the increased intraabdominal pressure and abdominal compartmentalization allowed for appropriate care for the cat in this case and a successful outcome (18, 21).

Urethral obstruction in felines is common, and thus, injury to the urethra can occur due to iatrogenic trauma such as catheterization (20). In this particular case, the trauma was not discovered until after permanent urinary diversion by perineal urethrostomy likely due to the size and location of the rent initially. Because of the cranial positioning of the defect as identified on the positive-contrast cystourethrogram and body condition score of the cat (8/9), the decision to perform the primary repair of the rent was elected and completed successfully as obesity can play a role in persistent abdominal compartmental syndrome (18). Seven days postoperatively, a repeat positive-contrast cystourethrogram was performed revealing no persistent tear. Short- and long-term outcomes of 2 and 8 weeks demonstrate the success of this revision. This report highlights the ability not only to identify a persistent tear but also to repair and manage one successfully in a cat, which has never been documented before. It describes a technique that could be pursued if other surgical revisions are not feasible.

## Data Availability

The raw data supporting the conclusions of this article will be made available by the authors, without undue reservation.
